# Effects of Secondary Hyperparathyroidism Treatment on Improvement in Anemia: Results from the MBD-5D Study

**DOI:** 10.1371/journal.pone.0164865

**Published:** 2016-10-20

**Authors:** Motoko Tanaka, Kazuki Yoshida, Shingo Fukuma, Kazuko Ito, Kazutaka Matsushita, Masafumi Fukagawa, Shunichi Fukuhara, Tadao Akizawa

**Affiliations:** 1 Department of Nephrology, Akebono Clinic, Kumamoto, Japan; 2 Departments of Epidemiology & Biostatistics, Harvard T.H. Chan School of Public Health, Boston, Massachusettes, United States of America; 3 Institute for Health Outcomes and Process Evaluation Research (iHope International), Kyoto, Japan; 4 Department of Healthcare Epidemiology, Graduate School of Medicine and Public Health, Kyoto University, Kyoto, Japan; 5 Division of Nephrology, Endocrinology and Metabolism, Tokai University School of Medicine, Isehara, Japan; 6 Department of Innovative Research and Education for Clinicians and Trainees (DiRECT), Fukushima Medical University Hospital, Fukushima, Japan; 7 Division of Nephrology, Department of Medicine, Showa University School of Medicine, Tokyo, Japan; Tokushima University Graduate School, JAPAN

## Abstract

**Objectives:**

Anemia is an important prognostic factor in hemodialysis patients. It has been reported that parathyroidectomy ameliorates anemia and reduces the requirement of postoperative erythropoiesis-stimulating agents. The objective of this study was to assess the effect of cinacalcet, which is considered as a pharmacological parathyroidectomy, on anemia in hemodialysis patients.

**Methods:**

We used data from a prospective cohort of Japanese hemodialysis patients with secondary hyperparathyroidism; the criteria were: intact parathyroid hormone concentrations ≥ 180 pg/mL or use of an intravenous or oral vitamin D receptor activator. All patients were cinacalcet-naïve at study enrollment. The main outcome measure was achievement of the target hemoglobin level (≥10.0 g/dL), which was measured repeatedly every 6 months. Cinacalcet exposure was defined as cumulative time since initiation. Both conventional longitudinal models and marginal structural models were adjusted for confounding factors.

**Results:**

Among 3,201 cinacalcet-naïve individuals at baseline, cinacalcet was initiated in 1,337 individuals during the follow up. Cinacalcet users were slightly younger; included more patients with chronic glomerulonephritis and fewer with diabetes; were more likely to have a history of parathyroidectomy; and were more often on activated vitamin D agents, phosphate binders, and iron supplements. After adjusting for both time-invariant and time-varying potential confounders, including demographics, comorbidities, comedications, and laboratory values, each additional 6-month duration on cinacalcet was associated with a 1.1-fold increase in the odds of achieving the target hemoglobin level.

**Conclusions:**

Cinacalcet may improve anemia in chronic hemodialysis patients with secondary hyperparathyroidism, possibly through pathways both within and outside the parathyroid hormone pathways. Further investigations are warranted to delineate the roles of cinacalcet not only in the management of chronic kidney disease–mineral and bone disorder but also in anemia control.

## Introduction

Anemia is an important complication of chronic maintenance hemodialysis and is known to be an important predictor of poor prognosis[1,2]. Many factors contribute to anemia in dialysis patients, such as decreased production of erythropoietin, resistance to erythropoietin, shortened survival of red blood cells (RBC), and diminished erythropoiesis[3–5]. Additionally, secondary hyperparathyroidism (SHPT), a major component of chronic kidney disease–mineral and bone disorder (CKD–MBD), is known to result in resistance to erythropoietin; shortened survival of RBC via the accumulation of high levels of parathyroid hormone (PTH), a uremic substance; and decreased hematopoiesis due to myelofibrosis, which partially explains the pathways linking CKD and anemia[5,6].

It has been reported that parathyroidectomy ameliorates anemia and reduces the requirement for postoperative erythropoiesis-stimulating agents. In recent years, vitamin D receptor activators (VDRA) have been shown to improve anemia in chronic hemodialysis patients through pleiotropic effects on the immune system, inflammation, and nutritional status, in addition to the SHPT-related pathways[7–9]. Similar studies have been conducted for the newly marketed cinacalcet, a calcimimetic drug used to treat SHPT, which is considered to be a form of pharmacological parathyroidectomy[10,11].

However, the relationship between cinacalcet use and improvement in anemia has not been clarified in a larger scale longitudinal study. We hypothesized that cinacalcet initiators (patients who initiate cinacalcet) would experience better anemia control (hemoglobin ≥ 10.0 g/dL) comapred with that experienced by cinacalcet non-initiators (those who do not initiate cinacalcet), after adjusting for both baseline and time-varying confounders. Thus, we conducted a longitudinal analysis of a large cohort of hemodialysis patients with SHPT in daily clinical practice to investigate the effect of cinacalcet on improvement of anemia.

## Materials and Methods

### Data Source

The Mineral and Bone Disorder Outcomes Study for Japanese Chronic Kidney Disease Stage 5D Patients (MBD-5D) was a 3-year, multicenter, prospective case-cohort, and cohort study of hemodialysis patients with SHPT. The eligibility criteria were as follows: (i) under hemodialysis at a participating facility as of January 1, 2008 and (ii) intact PTH (iPTH) concentrations ≥ 180 pg/mL or receipt of intravenous VDRA (calcitriol or maxacalcitol) or oral active VDRA treatment (falecalcitriol, the only oral VDRA approved in Japan for SHPT treatment at the time of study conception). Details of the study design have been described previously[12]. Most data were collected at 3- or 6-month intervals. As hemoglobin was measured every 6 months, we utilized the 6-month interval data in this analysis. This study was approved by the Central Ethics Committee at the Kobe University School of Medicine. The study was conducted in accordance with the principles of the Declaration of Helsinki, and the MBD-5D study was registered at ClinicalTrials.gov (NCT00995163).

### Study Population

Only the patients in the predefined subcohort were included in this study. Patients with cancer or cancer-related comorbidity at the time of enrollment were excluded from the analysis; and if a patient was diagnosed with cancer during the observation period, data obtained post-diagnosis were not included in the analysis.

### Outcomes

The primary outcome of interest was achievement of the target hemoglobin level (≥10.0 g/dL). Hemoglobin levels were assessed as the secondary outcome. Both outcomes were repeatedly measured in each individual every 6 months, up to a maximum of six measurements.

### Exposure

Cinacalcet use was the exposure of interest. To emulate the intention-to-treat effect[13] in the randomized controlled trials, we defined a time-varying “ever exposed” status variable. That is, patients contributed “unexposed” observations until they initiated cinacalcet; and, once initiated, these patients always contributed “exposed” observations regardless of their current cinacalcet use status. This time-varying “ever exposed” status was then converted to a “time since initiation” variable to account for the dose response over time. As the study enrollment period was prior to the market approval of cinacalcet in Japan, all patients were cinacalcet-naïve at baseline, allowing for a clear definition of cinacalcet initiation (new user design).

### Covariates

Clinically relevant variables that could influence subsequent cinacalcet use and hemoglobin levels were considered to be confounders that required adjustment. These included demographics such as age and gender, baseline comorbidities such as the cause of CKD, smoking status, diabetes mellitus, heart disease, cerebrovascular disease, and duration of maintenance hemodialysis history. Any history of hyperparathyroidism treatment (parathyroidectomy, and ethanol or vitamin D injection therapy) was also assessed at baseline. Baseline-only laboratory tests included creatinine and total protein. Information on these variables was provided by each patient’s treating physician.

Comedications and laboratory test values were time-varying. Medications included intravenous or oral activated vitamin D, phosphate binders (either calcium-containing or otherwise), erythropoiesis-stimulating agents, and iron supplements. Time-varying laboratory test values included Kt/V, intact PTH (calculated from the whole PTH level using the equation iPTH = whole PTH × 1.7 as direct assays differed between study sites)[14,15], albumin-adjusted serum calcium (calcium–albumin–4.0)[16], serum phosphate, alkaline phosphatase, albumin, ferritin, iron, and hemoglobin.

To ensure the time-varying covariates, exposure, and outcome were correctly ordered, and also to avoid reverse causality (where the outcome precedes the covariates), the covariate values were obtained 6 months prior to the exposure, which in turn was ascertained 6 months prior to the outcome. In the sensitivity analysis, the lagged exposure status 12 months prior to the outcome was used to examine the potentially delayed effect of cinacalcet on hemoglobin.

### Statistical analysis

Continuous variables were expressed as means and standard deviations or as medians with 25^th^ and 75^th^ percentiles, as appropriate. Categorical variables were summarized as count and proportion. As the exposure varied over time, we present the baseline characteristics at the time of study enrollment separately for patients who initiated cinacalcet at some point during the follow-up and for those who did not start at any time during the follow-up. Group imbalance was examined with the standardized mean difference[17]; in general, a standardized mean difference greater than 0.1 was considered as indicating group imbalance. For clinically relevant variables required for the adjusted regression models, missing values were imputed with multiple imputation using the mice package in R[18]. Cinacalcet initiation over time was described using Kaplan–Meier estimation techniques. An unadjusted description of mean hemoglobin level was calculated at each time point for patients who started cinacalcet (“calcinet users,” in the intention-to-treat sense) and those who remained off cinacalcet (“non-users”).

In the regression analysis, the generalized estimating equation (GEE) model was used to account for the within-patient correlation of longitudinal, repeatedly measured outcomes. For the primary outcome of achievement of the target hemoglobin level (≥10 g/dL), the GEE longitudinal logistic model was used. For the secondary outcome of continuous hemoglobin level, the GEE longitudinal linear model was used.

Assessment of the effect of time-varying exposure was complicated by the presence of time-varying confounders, clinical characteristics influenced by previous treatment that affect the subsequent treatment choices and outcomes of interest[19]. These time-varying characteristics may therefore be potential confounders and mediators at the same time. To account for these variables, we employed two strategies: use of the conventional GEE model incorporating time-varying covariates, and use of a marginal structural model[19]. Marginal structural models with the inverse probability of treatment weighting (IPTW) estimator[19] are increasingly used to disentangle complex time-varying confounding because they can provide better confounding control in such a situation. Due to the longitudinal nature of the outcome data, we constructed a marginal structural model for the repeatedly measured outcomes[20].

An exploratory analysis among the cinacalcet users assessed changes in iPTH, calcium, and phosphate and hemoglobin over time. Also an ad hoc subgroup analysis based on the median iPTH value (265.20 pg/ml) was conducted to examine the influence of the baseline iPTH value at the study enrollment. All analyses were conducted with R version 3.3 (www.r-project.org) and its additional packages *tableone*, *mice*, *geepack*, and *survey*. Hypothesis tests were considered statistically significant when p < 0.05.

## Results

**Fig 1:**
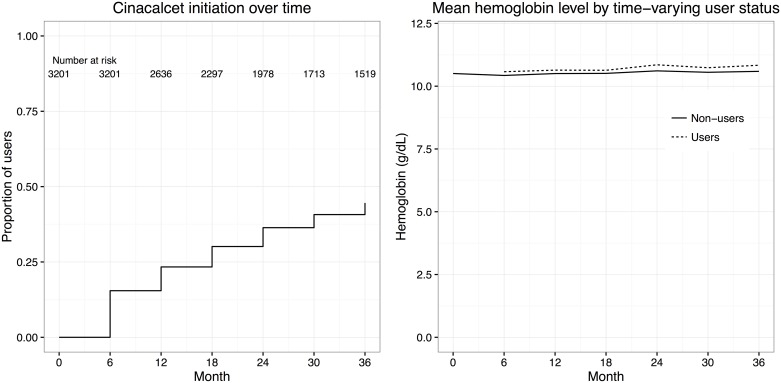
Cinacalcet initiation during the follow-up period, and mean hemoglobin level by time-varying cinacalcet status (user or non-user).

At the baseline, the registery included 3,201 chronic hemodialysis patients with secondary hyperparathyroidism. Among these, 1,337 patients initiated cinacalcet at some point during the follow-up (44.6% by the end of follow-up; Fig 1, **left panel**), constituting the initiators. The baseline characteristics of the future initiators (none of whom were on cinacalcet at enrollment) and the cinacalcet non-users are presented in Table 1. Compared with the non-users, the prospective calcinet users showed the following characteristics at their visit 6 months prior to cinacalcet initiation: younger age (mean 58.9 years vs 63.9 years), a greater likelihood of chronic glomerulonephritis being the reason for hemodialysis (53.3% chronic glomerulonephritis and 15.7% diabetic nephropathy vs 39.1% chronic glomerulonephritis and 30.1% diabetic nephropathy), longer duration of hemodialysis (median 10.9 years vs 5.8 years), more frequent surgical therapy for secondary hyperparathyroidism (10.8% vs 4.9%), more frequent vitamin D agent use (86.9% vs 73.8%), more frequent phosphate binder use (91.8% vs 81.6%), slightly more frequent erythropoietic agent use (71.4% vs 69.3%), slightly more frequent iron supplement use (19.7% vs 14.1%), higher adjusted calcium (mean 10.2 mg/dL vs 9.4 mg/dL), higher inorganic phosphate (mean 5.8 mg/dL vs 5.4 mg/dL), and slightly higher hemoglobin (10.65 g/dL vs 10.44 g/dL).

**Table 1:** Characteristics of the cinacalcet non-users at registry enrollment and of the cinacalcet initiators at the visit immediately prior to cinacalcet initiation.

	Non-users (enrollment)	Users (pre-initiation)	SMD
N	1864	1337	
**Age, years (mean (SD))**	63.86 (12.81)	58.88 (11.78)	0.405
**Male (%)**	1168 (62.7)	800 (59.8)	0.058
**Primary disease (%)**			0.400
** Chronic glomerulonephritis**	728 (39.1)	712 (53.3)	
** Diabetic nephropathy**	561 (30.1)	210 (15.7)	
** Chronic pyelonephritis**	31 (1.7)	25 (1.9)	
** Polycystic kidney**	67 (3.6)	72 (5.4)	
** Hypertensive nephrosclerosis**	130 (7.0)	58 (4.3)	
** Others**	347 (18.6)	260 (19.4)	
**Smoking (%)**	202 (10.8)	149 (11.1)	0.010
**Diabetes (%)**	715 (38.4)	282 (21.1)	0.385
**Coronary heart disease (%)**	499 (26.8)	298 (22.3)	0.104
**Congestive heart failure (%)**	158 (8.5)	80 (6.0)	0.096
**Cerebrovascular disease (%)**	113 (6.3)	58 (4.4)	0.082
**Chronic hemodialysis duration, years [median (IQR)]**	5.82 [2.53, 11.44]	10.94 [6.81, 16.84]	0.532
**History of surgical therapy (%)**	92 (4.9)	145 (10.8)	0.221
** Parathyroidectomy (%)**	84 (4.5)	107 (8.0)	0.145
** Ethanol injection (%)**	7 (0.4)	32 (2.5)	0.177
** Vitamin D injection (%)**	10 (0.6)	21 (1.7)	0.102
**Baseline creatinine, mg/dL [mean (SD)]**	10.74 (2.84)	11.97 (2.73)	0.442
**Baseline total protein, g/dL [mean (SD)]**	6.57 (0.55)	6.54 (0.53)	0.059
**IV or oral activated vitamin D use (%)**	1376 (73.8)	1162 (86.9)	0.334
**Total activated vitamin D dose Âμg/4 weeks**	4.00 [0.00, 7.00]	6.45 [3.50, 9.00]	0.412
**Phosphate binder use (%)**	1521 (81.6)	1227 (91.8)	0.303
**Erythropoietin use (%)**	1292 (69.3)	955 (71.4)	0.046
**Iron supplement use (%)**	263 (14.1)	263 (19.7)	0.149
**Body mass index [mean (SD)]**	21.35 (3.63)	21.35 (3.27)	<0.001
**Kt/V [median (IQR)]**	1.38 [1.20, 1.56]	1.46 [1.30, 1.62]	0.286
**Intact parathyroid hormone, pg/mL [median (IQR)]**	237.85 [185.50, 328.02]	340.00 [223.00, 506.60]	0.517
**Serum calcium, mg/dL [mean (SD)]**	9.13 (0.83)	9.98 (0.73)	1.094
**Adjusted serum calcium, mg/dL [mean (SD)]**	9.40 (0.95)	10.22 (0.85)	0.908
**Serum phosphate, mg/dL [mean (SD)]**	5.37 (1.35)	5.80 (1.32)	0.321
**Alkaline phosphatase, IU/L [median (IQR)]**	248.00 [190.00, 336.50]	256.00 [198.00, 346.00]	0.074
**Albumin, g/dL [mean (SD)]**	3.73 (0.37)	3.76 (0.35)	0.088
**Serum ferritin, ng/mL [mean (SD)]**	198.63 (365.08)	148.81 (209.23)	0.167
**Serum iron, μg/dL [median (IQR)]**	58.00 [44.00, 75.00]	59.00 [45.00, 76.00]	0.002
**Hemoglobin, g/dL [mean (SD)]**	10.44 (1.19)	10.65 (1.09)	0.182
**Hemoglobin < 10 g/dL (%)**	600 (32.2)	329 (24.6)	0.169

Abbreviations: SD: standard deviation; IQR: interquartile range; SMD: standardized mean difference

**Fig 2:**
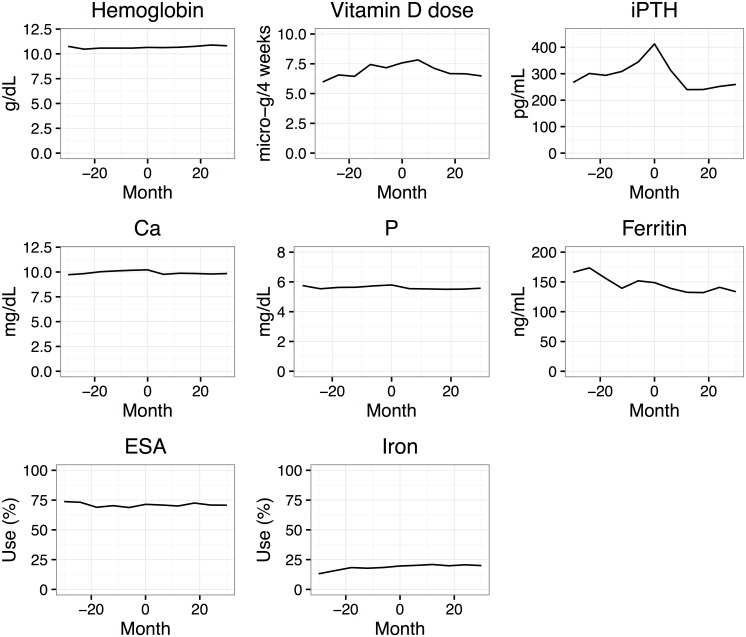
Trajectories of laboratory findings and medication use for the cinacalcet users before and after cinacalcet initiation.

The mean hemoglobin levels at each time point for those who had started cinacalcet by that time and for those who had not are plotted in Fig 1 (right panel). The hemoglobin levels were slightly higher at each time point for those patients who had started cinacalcet (Users) than for those who had remained off cinacalcet (Non-users), with the difference varying from 0.1 to 0.2 g/dL. As noted earlier, there were no cinacalcet users at the time of study enrollment. Changes in several variables before and after cinacalcet initiation are shown in Fig 2. As expected, iPTH increased steeply before cinacalcet initiation and decreased dramatically afterwards. Serum calcium and phosphate showed a modest decline after initiation. Activated vitamin D doses varied greatly between individuals; the mean value decreased after cinacalcet initiation. Erythropoiesis-stimulating agent use and iron supplement use were stable after initiation of cinacalcet (around 70% for erythropoiesis-stimulating agent and around 20% for iron supplement).

In the unadjusted GEE model (Table 2), the use of cinacalcet for 6 months was associated with a 1.1-fold increase in the odds of achieving the target hemoglobin level of ≥10 g/dL (odds ratio 1.13, 95% confidence interval [1.09, 1.17], p < 0.001). The mean change in hemoglobin level for each additional 6-month use of cinacalcet was +0.064 g/dL [0.047, 0.081], p < 0.001 (Table 3**)**.

**Table 2:** Unadjusted and adjusted effects of each 6 additional months of cinacalcet therapy on the odds of achieving the treatment target (intention-to-treat effect).

Hemoglobin ≥ 10 g/dl as a binary outcome	OR [95% CI]	p
**Generalized estimating equation**		
**Unadjusted**	1.13 [1.09, 1.17]	<0.001
**Fully adjusted**	1.08 [1.04, 1.13]	<0.001
		
**Inverse probability of treatment weight**		
**Fully adjusted**	1.11 [1.04, 1.20]	0.013

**Abbreviations**: OR: odds ratio; CI: confidence interval.

**Table 3:** Unadjusted and adjusted effects of each 6 additional months of cinacalcet therapy on hemoglobin levels (intention-to-treat effect).

Hemoglobin value as a continuous outcome	Change (g/dL) [95% CI]	p
**Generalized estimating equation**		
**Unadjusted**	0.064 [0.047, 0.081]	<0.001
**Fully adjusted**	0.042 [0.019, 0.066]	0.001
		
**Inverse probability of treatment weight**		
**Fully adjusted**	0.067 [0.020, 0.114]	0.02

**Abbreviations**: CI: confidence interval.

The adjusted models were controlled for age, sex, time since enrollment, primary cause of chronic kidney disease, smoking status, diabetes, ischemic heart disease, congestive heart failure, chronic hemodialysis duration, history of surgical therapy for hyperparathyroidism, baseline creatinine, baseline total protein, comedications (activated vitamin D, phosphate binders, erythropoietin-stimulating agents, iron supplements), and laboratory measures (Kt/V, intact parathyroid hormone, albumin-adjusted serum calcium, phosphate, alkaline phosphatase, albumin, ferritin, serum iron, and hemoglobin level at the previous visit). The adjusted regression results for the binary outcome of achievement of target hemoglobin level are presented in Table 2. The conventional adjusted GEE model showed an adjusted odds ratio for each additional 6-month use of cinacalcet of 1.08 (1.04, 1.13), p < 0.001. The IPTW-based marginal structural model (Table 2) adjusting for the same set of variables gave a similar result for the odds of achieving the target hemoglobin level [adjusted odds ratio 1.11 (1.04, 1.20), p = 0.013].

The adjusted regression results for the outcome of continuous mean hemoglobin levels are presented in Table 3. The conventional adjusted GEE model showed an adjusted effect per each additional 6 months of cinacalcet use of +0.042 g/dL (0.019, 0.066), p = 0.001. The IPTW-based marginal structural model gave a slightly higher result [adjusted mean difference +0.067 g/dL (0.020, 0.114), p = 0.020].

In the exploratory analysis among the cinacalcet users, the hemoglobin trajectories were most clearly differentiated throughout the study period between those whose phosphate level had increased and those whose level had declined. The trajectories were not as clearly separated by similar differences in iPTH or calcium levels (S1 Fig). In the subgroup analysis, those who had higher-than-median baseline iPTH had numerically higher improvement in anemia than those who had lower-than-median baseline iPTH (adjusted odds ratio 1.09 (1.04, 1.14) vs 1.07 (1.00, 1.15) in GEE; adjusted odds ratio 1.14 (1.05, 1.25) vs 1.07 (0.99, 1.17) in IPTW), however, the group difference did not reach statistical significance (p = 0.657 for GEE; p = 0.304 for IPTW).

## Discussion

In order to clarify the effect of cinacalcet use on the anemia control status, we conducted an analysis of the MBD-5D prospective cohort of chronic hemodialysis patients. We found that each additional 6 months on cinacalcet was associated with an approximately 1.1-fold increase in the odds of maintaining the anemia control target of hemoglobin ≥ 10 g/dL. When measured on the continuous mean hemoglobin-level scale, we also found that each additional 6 months on cinacalcet was associated with a hemoglobin increment of 0.042–0.067 g/dL. These findings suggest the potential benefit of cinacalcet use for the control of anemia in chronic hemodialysis patients with SHPT.

SHPT is known to be one of the pathological conditions that cause anemia[21,22], and SHPT and anemia coexist in most hemodialysis patients. Indeed, as many as 70% of the present study subjects had anemia and were receiving an erythropoietin-simulating agent. However, in routine clinical settings SHPT and anemia are often managed separately and from different viewpoints. When physicians manage anemia only by controlling the hemoglobin level with erythropoietin-simulating agents, few would notice whether the use of cinacalcet improved the anemia. The association between cinacalcet use and improvement of anemia in the present study suggests that it could be possible to manage SHPT and anemia simultaneously in an integrated manner.

Our findings are consistent with the hypothesis, based on the findings of basic science papers[5,23], that blockage of high parathyroid hormone levels may improve anemia in patients with secondary hyperparathyroidism. Although non-significant due to reduced sample sizes, the exploratory subgroup analysis by the baseline iPTH demonstrated numerically higher effects in those who had higher-than-median baseline iPTH levels. SHPT is known to cause anemia via several possible pathways. Erythrocyte lifespans decrease in response to high PTH levels[24]; and high PTH levels inhibit hematopoietic stem cell activity, particularly that of erythroid burst-forming units, as well as inducing myelofibrosis and contributing to erythropoietin resistance[24,25].

Curative parathyroidectomy has been reported to improve myelofibrosis in patients with *primary* hyperparathyroidism, thereby improving anemia[26,27]. Another retrospective report has described the potential benefits of parathyroidectomy in *secondary* hyperparathyroidism[28]. The authors reported that, after parathyroidectomy, the use of erythropoietin-stimulating agents was rendered unnecessary. These findings suggest that parathyroidectomy induces a reduction in PTH levels and an improved resistance to vitamin D, thereby inducing improvements in anemia and erythropoietin resistance. This deduction is in accordance with the findings of the present study regarding improvement of anemia with cinacalcet, which is considered to be a form of “medical parathyroidectomy.”

Cinacalcet is also likely to participate in improving anemia via pathways not driven by PTH levels. Calcium-sensing receptors play a role in the homing of hematopoietic stem cells to bone marrow niches as well as in engraftment promotion[29]. The allosteric action of cinacalcet is likely to act against the calcium-sensing receptors on hematopoietic stem cells, thus contributing to anemia improvement. Recently, Koizumi et al.[30] reported that administration of cinacalcet reduced the serum level of fibroblast growth factor 23 in patients with SHPT. Similar data were also obtained from the EVOLVE study(31). Also, Coe et al.[31] found that loss of fibroblast growth factor 23 increased erythrocyte production in mice. Therefore, in addition to reducing PTH, reduction of fibroblast growth factor 23 may be one pathway through which cinacalcet improves anemia.

Our study is not without limitations. As we utilized a cohort of SHPT patients (from the MBD-5D study), the generalizability of our findings beyond this type of patient (i.e., chronic hemodialysis patients in general) is unknown. As with any observational studies, problems with unmeasured (or unmeasurable) confounders cannot be ruled out. The present study did not record the dosage of comedications that could influence hemoglobin levels (erythropoiesis-simulating agents and iron supplements), the patients’ activities of daily living, their nutritional intake status, or inflammatory markers. However, these factors are less likely to affect the decision to initiate cinacalcet (the exposure). Outcome predictors that are not strongly associated with determining exposure have only limited potential to be confounders[32]. Usage of erythropoiesis-stimulating agents and iron supplements did not change after cinacalcet initiation. Thus, we could speculate that their dosage probably did not change dramatically. The mean ferritin level decreased after cinacalcet initiation, thus, adjustment in anemia treatment may have benefited these patients further.

Our study had several important strengths. The MBD-5D cohort is a large-scale prospective cohort of chronic hemodialysis patients, which has collected a rich set of data at regular intervals, allowing assessment of time-varying exposure and the control of time-dependent confounding. The cohort’s focus on SHPT patients can be considered as its strength. It allowed us to analyze a cohort of patients with relatively similar backgrounds. The enrollment period of the cohort was prior to the market approval of cinacalcet in Japan; thus, no patients were on cinacalcet at baseline. The new user design is generally recommended in medication studies[33], as it allows clearer distinction of what prompted medication use, and what resulted from this. We employed two types of statistical analysis to adjust for time-dependent confounding, namely the conventional longitudinal GEE model and the IPTW-based marginal structural model. In this instance, we found relatively similar results from both analyses. The subtle differences are likely to be due to the handling of covariates after cinacalcet initiation.

In conclusion, we found that each additional 6 months on cinacalcet was associated with a 1.1-fold increase in the odds of achieving the hemoglobin control target of ≥10 g/dL in chronic hemodialysis patients with secondary hyperparathyroidism. However, the increment of the increase in hemoglobin was relatively small, and further investigations are warranted to define the roles of cinacalcet not only in CKD-MBD management but also in anemia control.

## Supporting Information

S1 Fig Hemoglobin trajectories among cinacalcet initiators. The users are grouped according to whether there was a decline or increase in the laboratory findings.
(TIFF)

S1 Table Unadjusted and adjusted effects of each 6 additional months of cinacalcet therapy on the odds of achieving the treatment target (12-month lagged outcome). (DOCX)

S2 Table Unadjusted and adjusted effects of each 6 additional months of cinacalcet therapy on hemoglobin levels (12-month lagged outcome). (DOCX)

## References

[1] Khan S, Pereira BJ. Hematocrit level associated mortality in hemodialysis patients, by Ma JZ, Ebben J, Xia H, Collins AJ. J Am Soc Nephrol 10:610–619, 1999. Semin Dial. 2000;13: 112–113. 10795115 10.1046/j.1525-139x.2000.00036.x

[2] Xia H, Ebben J, Ma JZ, Collins AJ. Hematocrit levels and hospitalization risks in hemodialysis patients. J Am Soc Nephrol. 1999;10: 1309–1316. 10361870 10.1681/ASN.V1061309

[3] Nissenson AR, Nimer SD, Wolcott DL. Recombinant human erythropoietin and renal anemia: molecular biology, clinical efficacy, and nervous system effects. Ann Intern Med. 1991;114: 402–416. 1992884 10.7326/0003-4819-114-5-402

[4] Ly J, Marticorena R, Donnelly S. Red blood cell survival in chronic renal failure. Am J Kidney Dis. 2004;44: 715–719. 15384023

[5] Rao DS, Shih MS, Mohini R. Effect of serum parathyroid hormone and bone marrow fibrosis on the response to erythropoietin in uremia. N Engl J Med. 1993;328: 171–175. 10.1056/NEJM199301213280304 8417383

[6] Neves PL, Triviño J, Casaubon F, Santos V, Mendes P, Romão P, et al Elderly patients on chronic hemodialysis with hyperparathyroidism: increase of hemoglobin level after intravenous calcitriol. Int Urol Nephrol. 2006;38: 175–177. 10.1007/s11255-004-1563-0 16502078

[7] Aucella F, Scalzulli RP, Gatta G, Vigilante M, Carella AM, Stallone C. Calcitriol increases burst-forming unit-erythroid proliferation in chronic renal failure. A synergistic effect with r-HuEpo. Nephron Clin Pract. 2003;95: c121–127. doi: 74837 14694273 10.1159/000074837

[8] Türk S, Akbulut M, Yildiz A, Gürbilek M, Gönen S, Tombul Z, et al Comparative effect of oral pulse and intravenous calcitriol treatment in hemodialysis patients: the effect on serum IL-1 and IL-6 levels and bone mineral density. Nephron. 2002;90: 188–194. 11818704 10.1159/000049041

[9] Keithi-Reddy SR, Addabbo F, Patel TV, Mittal BV, Goligorsky MS, Singh AK. Association of anemia and erythropoiesis stimulating agents with inflammatory biomarkers in chronic kidney disease. Kidney Int. 2008;74: 782–790. 10.1038/ki.2008.245 18547996 PMC2739585

[10] Battistella M, Richardson RMA, Bargman JM, Chan CT. Improved parathyroid hormone control by cinacalcet is associated with reduction in darbepoetin requirement in patients with end-stage renal disease. Clin Nephrol. 2011;76: 99–103. 21762640 10.5414/cn106640

[11] Mpio I, Boumendjel N, Karaaslan H, Arkouche W, Lenz A, Cardozo C, et al [Secondary hyperparathyroidism and anemia. Effects of a calcimimetic on the control of anemia in chronic hemodialysed patients. Pilot Study]. Nephrol Ther. 2011;7: 229–236. 10.1016/j.nephro.2011.01.008 21353659

[12] Fukuhara S, Akizawa T, Fukagawa M, Onishi Y, Yamaguchi T, Hasegawa T, et al Mineral and bone disorders outcomes study for Japanese chronic kidney disease stage 5D patients: rationale and study design. Ther Apher Dial. 2011;15: 169–175. 10.1111/j.1744-9987.2010.00906.x 21426510

[13] Cole SR, Hernán MA, Margolick JB, Cohen MH, Robins JM. Marginal structural models for estimating the effect of highly active antiretroviral therapy initiation on CD4 cell count. Am J Epidemiol. 2005;162: 471–478. 10.1093/aje/kwi216 16076835

[14] Guideline Working Group, Japanese Society for Dialysis Therapy. Clinical practice guideline for the management of secondary hyperparathyroidism in chronic dialysis patients. Ther Apher Dial. 2008;12: 514–525. 10.1111/j.1744-9987.2008.00648.x 19140852

[15] Reichel H, Esser A, Roth H-J, Schmidt-Gayk H. Influence of PTH assay methodology on differential diagnosis of renal bone disease. Nephrol Dial Transplant. 2003;18: 759–768. 12637646 10.1093/ndt/gfg144

[16] Payne RB, Little AJ, Williams RB, Milner JR. Interpretation of serum calcium in patients with abnormal serum proteins. Br Med J. 1973;4: 643–646. 4758544 10.1136/bmj.4.5893.643 PMC1587636

[17] Austin PC. An Introduction to Propensity Score Methods for Reducing the Effects of Confounding in Observational Studies. Multivariate Behav Res. 2011;46: 399–424. 10.1080/00273171.2011.568786 21818162 PMC3144483

[18] Buuren van S, Groothuis-Oudshoorn K. mice: Multivariate Imputation by Chained Equations in R. Journal of Statistical Software. 2011;45: 1–67.

[19] Robins JM, Hernán MA, Brumback B. Marginal structural models and causal inference in epidemiology. Epidemiology. 2000;11: 550–560. 10955408 10.1097/00001648-200009000-00011

[20] Hernán MA, Brumback BA, Robins JM. Estimating the causal effect of zidovudine on CD4 count with a marginal structural model for repeated measures. Stat Med. 2002;21: 1689–1709. 10.1002/sim.1144 12111906

[21] Brancaccio D, Cozzolino M, Gallieni M. Hyperparathyroidism and anemia in uremic subjects: a combined therapeutic approach. J Am Soc Nephrol. 2004;15 Suppl 1: S21–24. 14684666 10.1097/01.asn.0000093369.09194.12

[22] Hörl WH. The clinical consequences of secondary hyperparathyroidism: focus on clinical outcomes. Nephrol Dial Transplant. 2004;19 Suppl 5: V2–8. 10.1093/ndt/gfh1049 15284353

[23] Massry SG. Pathogenesis of the anemia of uremia: role of secondary hyperparathyroidism. Kidney Int Suppl. 1983;16: S204–207. 6376915

[24] Bogin E, Massry SG, Levi J, Djaldeti M, Bristol G, Smith J. Effect of parathyroid hormone on osmotic fragility of human erythrocytes. J Clin Invest. 1982;69: 1017–1025. 10.1172/JCI110505 6281309 PMC370157

[25] Meytes D, Bogin E, Ma A, Dukes PP, Massry SG. Effect of parathyroid hormone on erythropoiesis. J Clin Invest. 1981;67: 1263–1269. 10.1172/JCI110154 7229028 PMC370692

[26] Bhadada SK, Sridhar S, Ahluwalia J, Bhansali A, Malhotra P, Behera A, et al Anemia and thrombocytopenia improves after curative parathyroidectomy in a patient of primary hyperparathyroidism (PHPT). J Clin Endocrinol Metab. 2012;97: 1420–1422. 10.1210/jc.2011-2845 22419706

[27] Bhadada SK, Bhansali A, Ahluwalia J, Chanukya GV, Behera A, Dutta P. Anaemia and marrow fibrosis in patients with primary hyperparathyroidism before and after curative parathyroidectomy. Clin Endocrinol (Oxf). 2009;70: 527–532. 10.1111/j.1365-2265.2008.03346.x 18727711

[28] Conzo G, Perna A, Della Pietra C, Esposito D, Nunziata A, Palazzo A, et al Role of parathyroidectomy on anemia control and erythropoiesis-stimulating agent need in secondary hyperparathyroidism of chronic kidney disease. A retrospective study in 30 hemodialysis patients. Ann Ital Chir. 2013;84: 25–31. 23047642

[29] Drüeke TB. Haematopoietic stem cells—role of calcium-sensing receptor in bone marrow homing. Nephrol Dial Transplant. 2006;21: 2072–2074. 10.1093/ndt/gfl206 16702207

[30] Moe SM, Chertow GM, Parfrey PS, Kubo Y, Block GA, Correa-Rotter R, et al Cinacalcet, Fibroblast Growth Factor-23, and Cardiovascular Disease in Hemodialysis: The Evaluation of Cinacalcet HCl Therapy to Lower Cardiovascular Events (EVOLVE) Trial. Circulation. 2015;132: 27–39. 10.1161/CIRCULATIONAHA.114.013876 26059012

[31] Coe LM, Madathil SV, Casu C, Lanske B, Rivella S, Sitara D. FGF-23 is a negative regulator of prenatal and postnatal erythropoiesis. J Biol Chem. 2014;289: 9795–9810. 10.1074/jbc.M113.527150 24509850 PMC3975025

[32] Hernan MA, Robins JM. Causal Inference [Internet]. Chapman & Hall/CRC; 2016 Available: http://www.hsph.harvard.edu/miguel-hernan/causal-inference-book/.

[33] Ray WA. Evaluating medication effects outside of clinical trials: new-user designs. Am J Epidemiol. 2003;158: 915–920. 14585769 10.1093/aje/kwg231

