# Supplementary figures and images for: Effects of Secondary Hyperparathyroidism Treatment on Improvement in Anemia: Results from the MBD-5D Study
Source: PLoS One. 2016 Oct 20;11(10):e0164865. doi: 10.1371/journal.pone.0164865 (PMC5072648; doi:10.1371/journal.pone.0164865)

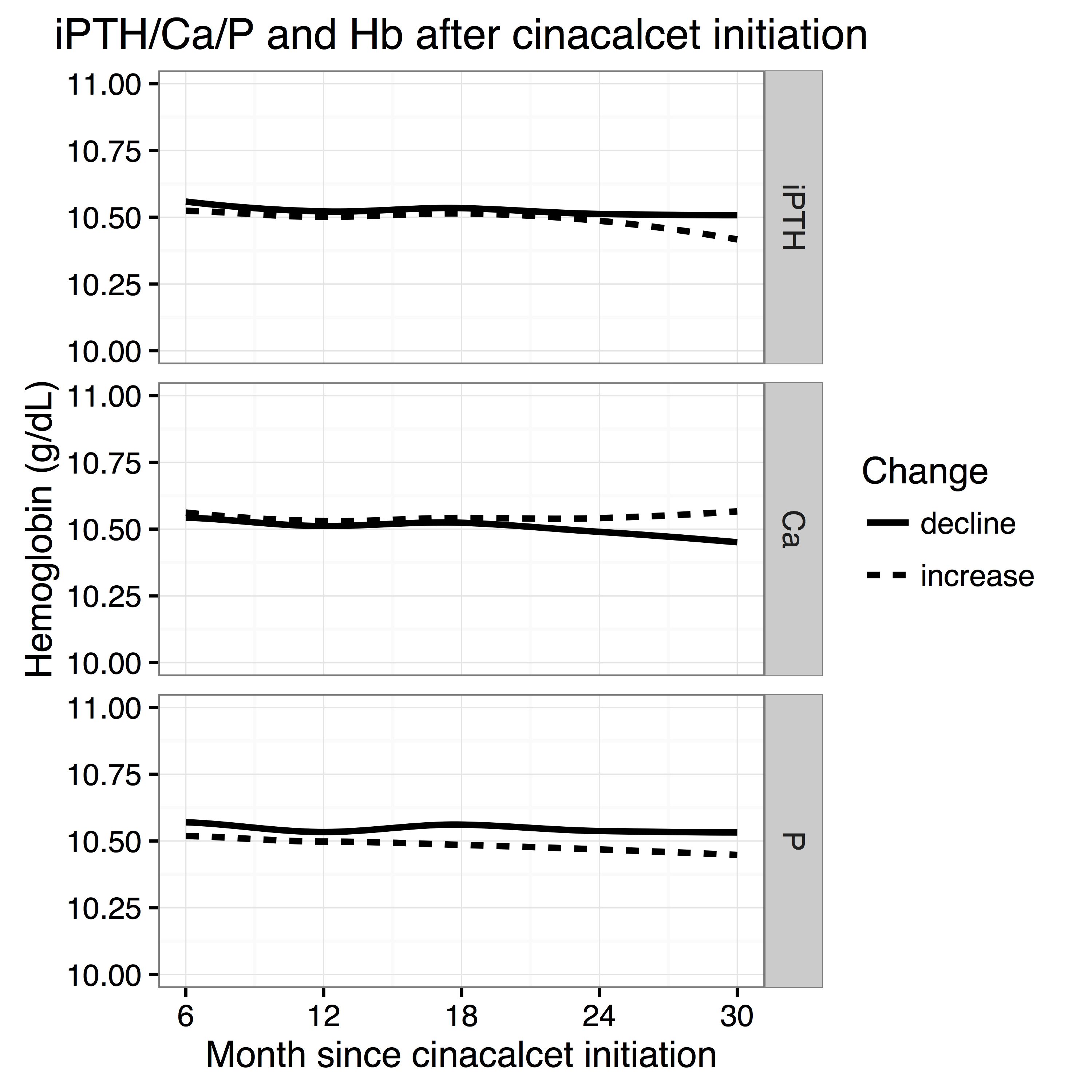

Supplement: S1 Fig — The users are grouped according to whether there was a decline or increase in the laboratory findings. (TIFF) [file pone.0164865.s001.tiff]
