# Supplementary material for: Effects of Secondary Hyperparathyroidism Treatment on Improvement in Anemia: Results from the MBD-5D Study
Source: PLoS One. 2016 Oct 20;11(10):e0164865. doi: 10.1371/journal.pone.0164865 (PMC5072648; doi:10.1371/journal.pone.0164865)
Supplement: S1 Table — (DOCX) [file pone.0164865.s002.docx]

**S1 Table**. Unadjusted and adjusted effects of each 6 additional months of cinacalcet therapy on the odds of achieving the treatment target (12-month lagged outcome).

| Hemoglobin ≥ 10 g/dl as a binary outcome | OR [95% CI] | p |
| --- | --- | --- |
| Generalized estimating equation |  |  |
| Unadjusted | 1.17 [1.12, 1.22] | <0.001 |
| Fully adjusted | 1.08 [1.02, 1.15] | 0.006 |
|  |  |  |
| Inverse probability of treatment weight |  |  |
| Fully adjusted | 1.15 [1.07, 1.24] | 0.002 |

**Abbreviations**: OR: odds ratio; CI: confidence interval.
