# Supplementary material for: Effects of Secondary Hyperparathyroidism Treatment on Improvement in Anemia: Results from the MBD-5D Study
Source: PLoS One. 2016 Oct 20;11(10):e0164865. doi: 10.1371/journal.pone.0164865 (PMC5072648; doi:10.1371/journal.pone.0164865)
Supplement: S2 Table — (DOCX) [file pone.0164865.s003.docx]

**S2 Table**. Unadjusted and adjusted effects of each 6 additional months of cinacalcet therapy on hemoglobin levels (12-month lagged outcome).

| Hemoglobin value as a continuous outcome | Change (g/dL) [95% CI] | p |
| --- | --- | --- |
| Generalized estimating equation |  |  |
| Unadjusted | 0.081 [0.059, 0.103] | <0.001 |
| Fully adjusted | 0.047 [0.014, 0.080] | 0.007 |
|  |  |  |
| Inverse probability of treatment weight |  |  |
| Fully adjusted | 0.073 [0.029, 0.117] | 0.006 |

**Abbreviations**: CI: confidence interval.
